# Case Report: Metastatic lung cancer in Meckel's Cave: a radiotherapy successful case and literature review

**DOI:** 10.3389/fonc.2025.1613711

**Published:** 2025-06-19

**Authors:** Chao Chen, Zhiqin Lu, Gang Lin, Yujie Hu, Yong Guo

**Affiliations:** ^1^ The First Clinical Medical College, Zhejiang Chinese Medical University, Hangzhou, China; ^2^ Department of Radiotherapy, The First Affiliated Hospital of Zhejiang Chinese Medical University (Zhejiang Provincial Hospital of Traditional Chinese Medicine), Hangzhou, China; ^3^ Department of Oncology, The First Affiliated Hospital of Zhejiang Chinese Medical University (Zhejiang Provincial Hospital of Traditional Chinese Medicine), Hangzhou, China

**Keywords:** Meckel’s cave metastasis, trigeminal cave metastasis, lung cancer, stereotactic radiosurgery, case report

## Abstract

**Background:**

Meckel’s cave (MC) is a highly uncommon site for metastatic disease, particularly from primary lung cancer.

**Case presentation:**

We report a clinical case of a 70-year-old man presenting with left trigeminal pain, left ptosis, and restricted abduction of the left eyeball. The patient had a 2-year history of stage IV lung squamous cell carcinoma. Contrast-enhanced brain MRI and FDG-PET/CT showed an ill-defined mass with a heterogeneously enhancing lesion involving the left MC and middle cranial fossa. Stereotactic radiosurgery (SRS, 30 Gy/5 fx) achieved significant improvement in symptoms and regression of radiologic tumors within 1 month. Our review of relevant literature identified only two reported cases of lung adenocarcinoma metastasizing to MC. In addition, we examined the limited literature on other malignant tumors metastatic to MC managed with radiotherapy.

**Conclusion:**

This is the first reported case of MC metastasis from lung squamous cell carcinoma successfully treated with SRS. Effective management of MC metastasis requires histology-specific radiotherapy strategies, with squamous cell carcinoma benefiting from hypofractionated SRS.

## Introduction

Lung cancer remains the leading cause of cancer-related mortality worldwide, with 20%–65% of patients developing brain metastases during disease progression (1, 2). The prognosis for these patients is poor, with a typical median survival of 1–2 months (3).

Meckel’s cave (MC), a dural recess that houses the trigeminal ganglion, is located at the petrous apex within the middle cranial fossa. This anatomically complex region is adjacent to critical neurovascular structures, including the cavernous sinus, internal carotid artery, and brainstem (4). Tumors originating in MC are rare, most commonly being trigeminal schwannomas and meningiomas (5). Metastases to MC account for merely 0.2% of cases, with common primary sites including lung, breast, prostate, and head/neck malignancies (6). Surgical resection, radiation therapy, and chemotherapy are the most common treatment methods. We present the first documented case of MC metastasis from squamous cell lung carcinoma managed with stereotactic radiosurgery (SRS). A systematic literature review contextualizes this rare entity, synthesizing evidence to optimize therapeutic decision-making.

## Case presentation

**Figure 1:**
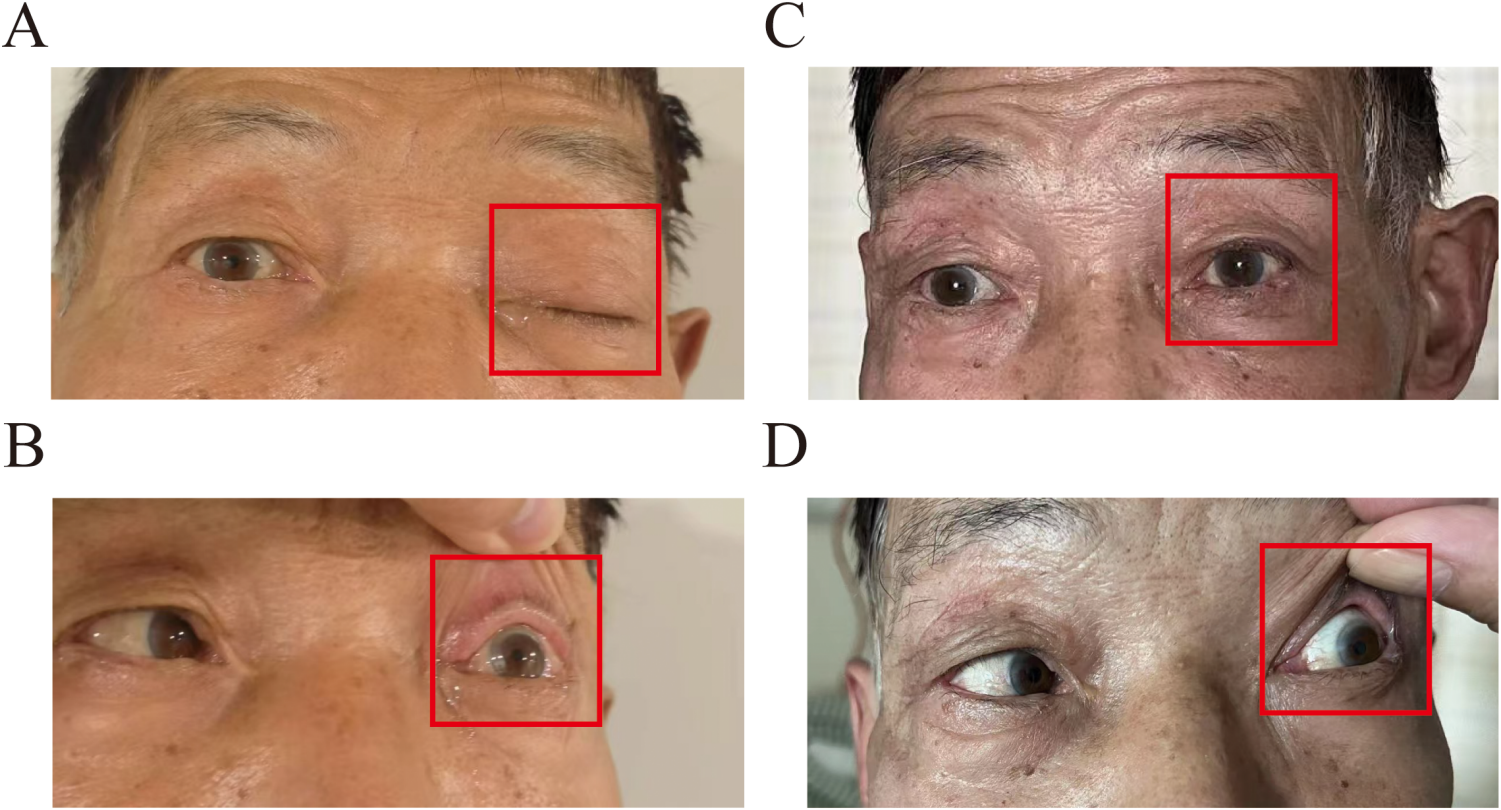
Clinical appearance at the initial examination: **(A)** labeled left upper eyelid ptosis (red box); **(B)** impaired left ocular abduction with limited lateral gaze (red box). Post-SRS treatment at 1 month: **(C)** labeled left upper eyelid ptosis returned to normal (red box); **(D)** improved left eye abduction impairment.

A 70-year-old man presented to our hospital with left-sided trigeminal pain. Physical examination revealed left ptosis (**Figure 1A**) and restricted abduction of the left eyeball (**Figure 1B**). The patient had a confirmed 2-year history of stage IVA squamous cell carcinoma of the right upper lung lobe, diagnosed via CT-guided biopsy. Genomic profiling of tumor tissue demonstrated: a pathogenic BRCA2 variant (c.8537_8538delAG, ClinVar ID 56199), a high tumor mutational burden (13.2 mutations/megabase); PD-L1 expression of 50% (22C3 pharmDx assay). First-line therapy consisted of nine cycles of tislelizumab monotherapy (200 mg IV q3w). Nine months later, he experienced oligoprogression in the mediastinal lymph nodes. In the same month, he underwent intensity-modulated radiotherapy (IMRT) with six MV X-rays for the mediastinal lymph node metastasis, receiving a dose of 60 Gy in 30 fractions, with satisfactory results. The patient became lost to follow-up posttreatment due to COVID-19.

**Figure 2:**
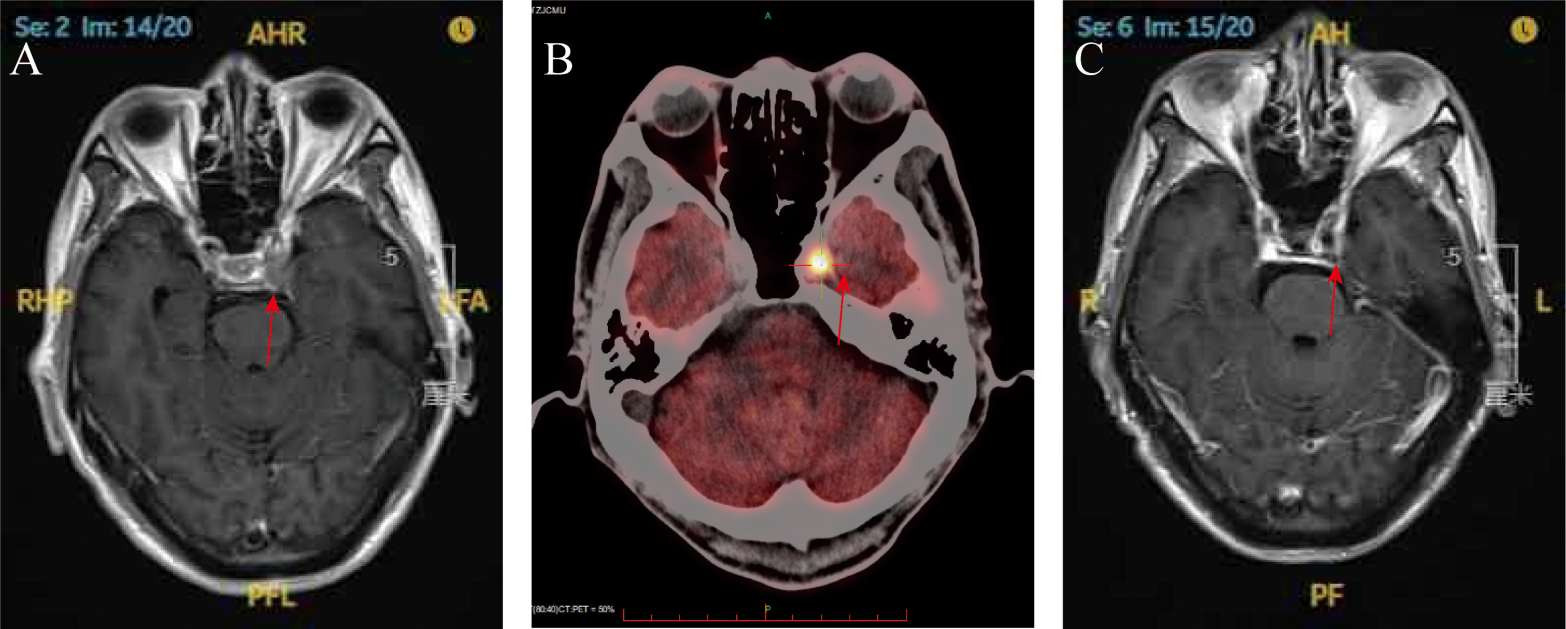
**(A)** Brain magnetic resonance imaging (T1-weighted with gadolinium enhancement) at the time of diagnosis (red arrow); **(B)** PET scan demonstrating significantly increased FDG uptake within the left Meckel’s cave at the time of diagnosis (red arrow); **(C)** brain magnetic resonance imaging (T1-weighted gadolinium enhancement) at 1-month post-SRS (red arrow).

**Figure 3:**
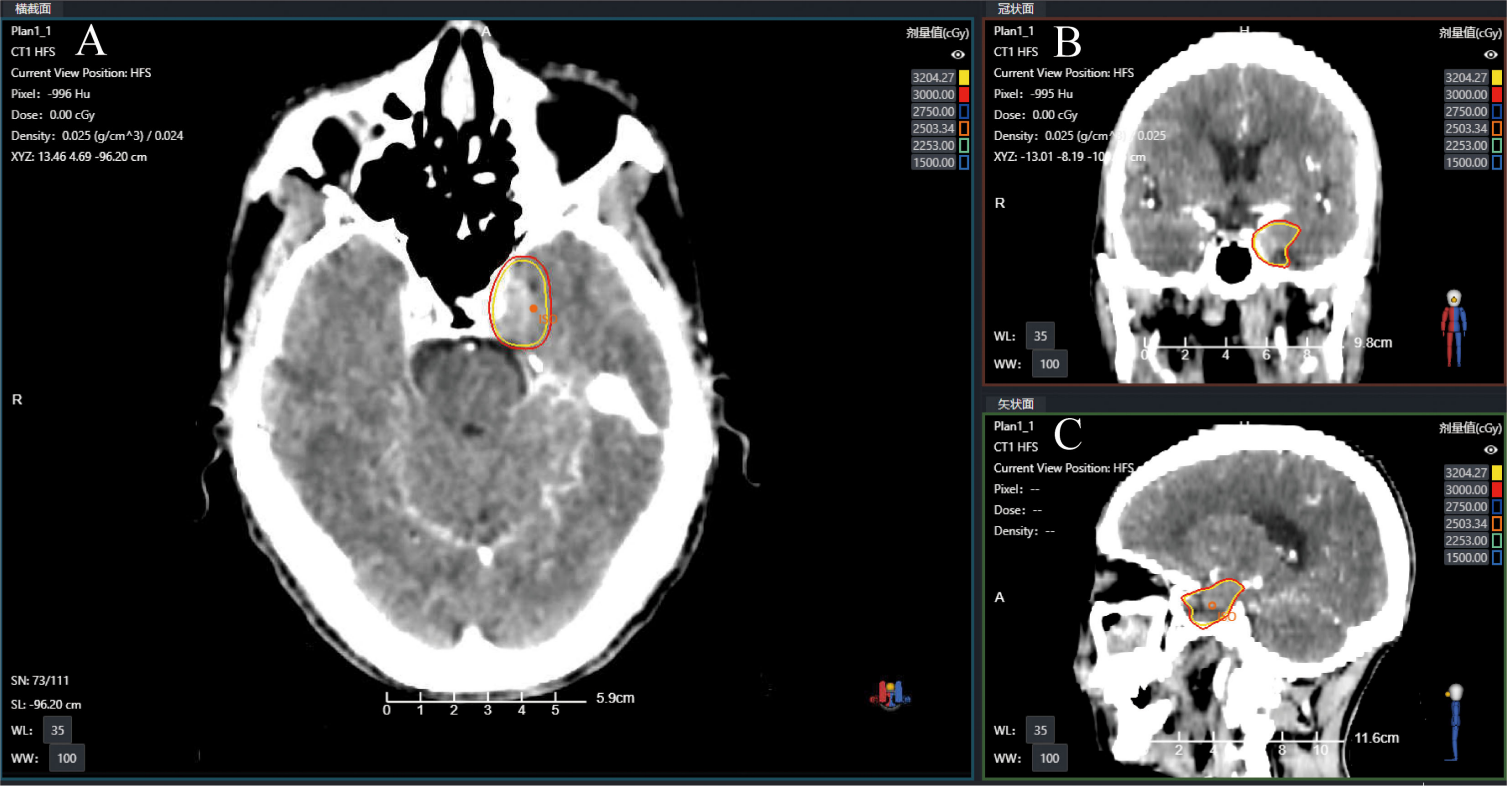
Radiotherapy treatment planning CT images showing the target with isodose lines in **(A)** axial **(B)** coronal, and **(C)** sagittal views.

Gadolinium-enhanced brain magnetic resonance imaging (MRI) (**Figure 2A**) and fluorodeoxyglucose positron emission tomography/computed tomography (FDG-PET/CT) (**Figure 2B**) demonstrated a 10 × 8 mm heterogeneously enhancing lesion (SUV max = 11.7) involving the left middle cranial fossa, with temporal lobe infiltration and MC extension. No extracranial metastatic foci were identified on whole-body staging. Multidisciplinary tumor board consensus established a presumptive diagnosis of MC metastasis. The patient subsequently underwent SRS using six MV photons, delivered at 30 Gy in five fractions with 95% isodose coverage of the planning target volume (PTV) (**Figure 3**). Maintenance immunotherapy with tislelizumab (200 mg IV q3w) was continued post-SRS. One month follow-up after SRS showed marked symptomatic improvement of left trigeminal pain, complete resolution of ptosis (**Figure 1C** **),** and restoration of left eye abduction (**Figure 1D**). Radiological follow-up showed substantial tumor reduction on MRI (**Figure 2C**). At the 9-month follow-up, the patient maintains Karnofsky Performance Status 90 with controlled disease, though declines further imaging surveillance per advance directive.

## Discussion

Metastatic involvement of the MC remains clinically rare, with only two confirmed cases reported in the PubMed-indexed literature (7, 8). Our study documents the third globally reported case and provides the first evidence of SRS efficacy in this anatomically challenging region, achieving significant improvement in symptoms and regression of radiologic tumors at 9-month follow-up without radiation-induced complications (**Table 1**). Comparative analysis reveals distinct clinicopathological patterns: the epidermal growth factor receptor (EGFR)-mutant adenocarcinoma case achieved 11-month stability with osimertinib (7), reflecting targeted therapy sensitivity. In contrast, both EGFR-wild-type non-small cell lung carcinoma cases (including ours) required radiotherapy/SRS, suggesting histology-dependent therapeutic strategies. While all cases presented with trigeminal neuropathy, our patient uniquely developed ptosis—a potential indicator of cavernous sinus compression requiring urgent intervention. Palliative radiotherapy (30 Gy) in Case 1 (8) provided minimal symptom relief with rapid progression, whereas SRS (30 Gy in 1 week) achieved durable local control—emphasizing the critical dose fractionation criticality in MC lesions.

**Table 1:** Case report of Meckel’s cave metastasis in a patient with lung cancer.

Case	Age/sex	Histology	Key symptoms	Metastasis site	Treatment	Outcome/prognosis
Cerase (8)	69/M	Lung adenocarcinoma EGFR wild-type	Right cheek sensory loss, paresthesia	Right MC and CS of ipsilateral trigeminal nerve	Palliative RT (30 Gy/2 weeks)	No improvement in symptoms/death (some weeks)
Iwasaki (7)	89/F	Lung adenocarcinoma EGFR L858R mutation	Left facial numbness, left facial pain, left intraoral numbness. headache, anorexia	Left MC (trigeminal nerve)	Osimertinib (80 mg/day)	Symptom resolution, tumor regression/stable for 11 months
Our case	70/M	Lung squamous cell carcinoma	Left trigeminal pain + ptosis	Left MC	SRS (30 Gy/1 week)	Symptom resolution, tumor regression/stable for 9 months

MC, Meckel’s cave; CS, cavernous sinus; RT, radiotherapy; SRS, stereotactic radiosurgery.

MC tumors constitute merely 0.5% of intracranial neoplasms, predominantly comprising trigeminal schwannomas (33%) and meningiomas (9, 10). Metastatic lesions are exceptionally rare, with primary origins including pulmonary, mammary, and genitourinary malignancies (7–14). This rarity is attributable to the following key factors: (1) anatomic constraints: the deep-seated location of the MC, enveloped by the cavernous sinus and petrous internal carotid artery, creates a hemodynamic “watershed zone” with low endothelial permeability. This may impede circulating tumor cells from extravasating through the intact blood–nerve barrier (9); (2) flow dynamics: vertebrobasilar arterial flow preferentially directs metastases to the posterior fossa, while the MC’s venous drainage via the inferior petrosal sinus may filter larger tumor emboli. In this case, hematogenous spread was implicated based on: (a) PET/CT showing no contiguous skull base lesions or cerebrospinal fluid (CSF) pathway abnormalities; and (b) the solitary MC lesion with T2 hyperintensity, which favored hematogenous rather than perineural spread. CSF analysis was not performed due to the absence of meningeal signs, which constitutes a study limitation in excluding concurrent microscopic CSF dissemination. Although dural enhancement patterns cannot completely rule out microscopic CSF involvement, the absence of multifocal leptomeningeal disease on MRI strongly supports a diagnosis of bloodstream metastasis.

MC metastasis is a rare type of metastatic tumor in the middle cranial fossa of the skull base, with only 18 reported cases since 2000 (6). While trigeminal neuralgia and cranial neuropathies are characteristic (15), our patient’s ocular abduction impairment suggests cavernous sinus infiltration beyond the resolution of initial imaging—an underrecognized pitfall in deep-seated lesions. Diagnostically, the lesion’s T2 hyperintensity with heterogeneous enhancement deviated from classic squamous cell metastasis patterns (4), mimicking a schwannoma until PET/CT confirmed systemic malignancy. This discordance highlights that MC metastases may lack “typical” imaging hallmarks, necessitating multimodal verification (MRI + metabolic staging) to avoid diagnostic delays.

Treatment options for metastatic disease to the MC include surgical resection and radiation therapy (RT), which encompasses both conventional radiation therapy and SRS and/or systemic therapy (6, 7, 9, 11, 16, 17). Surgical resection of MC metastases can be complicated by the involvement of neurovascular structures, potentially increasing morbidity and mortality. Radiation therapy has been shown to be highly effective in alleviating cranial nerve deficits, particularly when administered early after symptom onset (9, 18). A retrospective study on SRS treatment for skull base tumors reported an average overall survival (OS) ranging from 1.8 to 16 months (18). However, patients presenting with cranial nerve palsy had a median survival of only 5 months (18). Prognosis is even more difficult to estimate for patients with tumor metastasis to the MC, with those with cranial nerve palsy often nearing the end of life. Given the complexity of surgery, patients’ comorbidities, and suboptimal functional status, surgical resection in this region carries a high risk of morbidity and mortality.

Radiotherapy remains a cornerstone in the management of MC metastases, particularly for lesions that are surgically challenging. To synthesize our case with eight previously reported cases (**Table 2**), a systematic search was conducted in PubMed, Embase, and Web of Science using the following keywords: (“Meckel’s cave” OR “trigeminal cave” OR “trigeminal ganglion”) AND (“metastasis” OR “metastases” OR “neoplasm seeding”) AND (“radiotherapy” OR “stereotactic radiosurgery” OR “SRS” OR “Gamma Knife”). The search was limited to studies published between January 2000 and February 2025 in English-language journals. Inclusion criteria were case reports or series describing metastatic lesions involving MC, with detailed documentation of clinical presentation, treatment modalities, and outcomes, and availability of full-text articles. Exclusion criteria included nonhuman studies; duplicate reports or conference abstracts lacking sufficient clinical data, and lesions originating from adjacent structures (e.g., meningiomas extending into MC). Two authors independently screened titles/abstracts, resolving discrepancies through consensus. Full texts of 21 potentially relevant articles were reviewed, with eight studies meeting the inclusion criteria (**Table 2**). We advocate a risk-adapted strategy: (1) histology-guided modality selection—for radioresistant tumors (renal cell carcinoma, melanoma), prioritize single-fraction SRS with doses of 20–24 Gy, followed by MRI surveillance every 3 months. Repeat SRS (cumulative dose ≤ 45 Gy) has been shown to provide durable control in recurrences, as demonstrated by Panizza et al. (16). In radiosensitive histologies (lung squamous cell carcinoma), hypofractionated SRS (30 Gy/5 fractions) optimizes trigeminal nerve preservation while maintaining tumoricidal effects, as demonstrated by our case’s 9-month progression-free survival. For EGFR/ALK-driven adenocarcinomas, initiate targeted therapy (e.g., osimertinib), with deferred SRS reserved for symptomatic progression (7); (2) neuroanatomical dose constraints include limiting the trigeminal nerve maximum dose (*D*_max_) to < 15 Gy in a single fraction, or the equivalent dose in 2 Gy fractions (EQD2 < 45 Gy, α/β = 2), to minimize post-SRS hypoesthesia. For lesions involving the cavernous sinus, maintain the optic pathway dose at ≤ 8 Gy to prevent radiation-induced neuropathy; (3) multidisciplinary integration requires whole-body FDG-PET/CT to exclude active extracranial disease before initiating local therapy. This paradigm emphasizes that optimal management of MC metastases demands precision radiotherapy tailored to histologic radiosensitivity, rigorous neuroanatomic dosimetry, and systemic disease control—principles validated by our case’s successful outcome.

**Table 2:** Radiotherapy outcomes for Meckel’s cave metastasis by primary histology.

Primary cancer	Cases (*n*)	Median OS (month)	Preferred RT approach	Key learning
Renal cell Ca (16, 17)	3	12	Repeat SRS (20–24 Gy/fx)	Cumulative dose ≤ 45 Gy safe
Lung squamous Ca	1 (ours)	9^*^	Hypofractionated SRS (30 Gy/5 fx)	Histology-specific efficacy
Prostate Ca (14)	1	3	Single-fraction SRS (22 Gy)	Rapid symptom relief
Hepatocellular (10)	1	0.5	Palliative WBRT	Avoid in radioresistant tumors
Melanoma (12)	1	12	SRS + immunotherapy	Synergy with immune checkpoint inhibitors

OS, overall survival; WBRT, whole-brain radiotherapy.

^*^PFS in our case.

To date, there have been no reported cases of lung cancer metastasizing to the skull base with the involvement of multiple cranial nerves. Our study has several limitations. First, histopathologic confirmation of the MC lesion was not obtained due to the high procedural risks associated with biopsy in this anatomically complex region, although imaging findings (contrast-enhanced MRI and FDG-PET/CT) and clinical progression strongly supported a metastatic etiology. Second, the follow-up period of 9 months limits the ability to draw definitive conclusions regarding the long-term efficacy of SRS and potential radiation-induced neurotoxicity, and no imaging improvement was documented. Finally, as a single-center case report, our findings require validation in larger cohorts to assess generalizability.

## Conclusions

This study reports the first documented case of MC metastasis from lung squamous cell carcinoma involving concurrent impairments of the oculomotor (III), trigeminal (V), and abducent (VI) nerves, suggesting early cavernous sinus infiltration due to multicranial neuropathy. The rarity of MC metastasis is attributed to the blood–nerve barrier limiting tumor cell extravasation and vertebrobasilar arterial flow preferentially directing metastases to the posterior fossa. Based on our findings and existing evidence, we propose that the management of MC metastasis should involve histology-specific radiotherapy strategies, with squamous cell carcinoma particularly benefiting from hypofractionated SRS (30 Gy in five fractions).

## Data Availability

The original contributions presented in the study are included in the article/supplementary material. Further inquiries can be directed to the corresponding author.
